# Predicting short-term blood pressure trajectories using lifestyle and behavioural factors: a prospective cohort study

**DOI:** 10.21542/gcsp.2026.24

**Published:** 2026-06-30

**Authors:** Merugu Sudhakar, Pradeep Dayanand, Sandeep Dayanand, Jasmin Martinez, Tridev Adak

**Affiliations:** 1 Rajiv Gandhi Institute of Medical Sciences, Adilabad, India; 2 St. Vincent Hospital, Erie, Pennsylvania, USA; 3 West Virginia University, Morgantown, West Virginia, USA; 4 Swargiya Dadasaheb Kalmegh Smruti Dental College & Hospital, Nagpur, Maharashtra, India

## Abstract

**Background:** Hypertension is a major contributor to cardiovascular morbidity and mortality. While lifestyle modification is central to prevention, evidence on short-term blood pressure (BP) dynamics in relation to behavioural factors remains limited.

**Objective:** To evaluate the influence of diet, physical activity, and psychological stress on systolic blood pressure (SBP) trajectories and to develop a regression-based model for short-term BP prediction.

**Methods:** In this 12-week prospective cohort study, 75 adults attending a preventive cardiology clinic were followed, with BP measured at baseline, 6 weeks, and 12 weeks using validated oscillometric methods. Lifestyle and behavioural factors were assessed using the Food Frequency Questionnaire (sodium intake and DASH adherence), IPAQ-SF (physical activity), and PSS-10 (stress). Linear mixed-effects regression identified predictors of SBP change, and Elastic Net regression predicted 12-week SBP.

**Results:** Sixty-seven participants (mean age 44.6 ± 10.8 years; 52.2% male) completed follow-up. Mean SBP declined from 134.2 ± 12.6 to 131.4 ± 11.5 mmHg. Higher physical activity and DASH adherence were associated with SBP reduction, whereas higher sodium intake and stress predicted SBP elevation (all *p* < 0.05). The Elastic Net model demonstrated strong predictive performance (*R*^2^ = 0.78; RMSE = 5.9 mmHg). Physical activity had a stronger effect in participants <45 years, and stress amplified sodium-related SBP increases.

**Conclusions:** Short-term SBP trajectories are strongly influenced by modifiable behavioural factors. Predictive models based on lifestyle data may support early risk stratification and personalized hypertension prevention.

## Introduction

Elevated blood pressure (BP) remains a leading global health challenge, contributing substantially to cardiovascular morbidity and mortality^1, 2^. As of 2024, an estimated 1.4 billion adults aged 30–79 years worldwide were living with hypertension, accounting for approximately 33% of the global population in this age group. Notably, two-thirds of these individuals resided in low- and middle-income countries, highlighting a substantial burden of disease in resource-limited settings^3^. Despite widespread recognition of the risks, BP control rates remain suboptimal in many settings, particularly in low- and middle-income countries, where detection, treatment and lifestyle support are often limited^4, 5^.

Given this backdrop, prevention and early identification of BP elevation has become a major public-health priority^6^. Alongside pharmacotherapy, multiple clinical guidelines recommend lifestyle modification—such as limiting dietary sodium, increasing physical activity, reducing excess weight and managing stress—as first-line strategies for BP control and cardiovascular risk reduction^7, 8^. Empirical and experimental evidence supports the efficacy of these interventions: for example, regular aerobic exercise and salt-reduction strategies have been shown to reduce systolic BP (SBP) by several mmHg in hypertensive and pre-hypertensive populations^9, 10^.

However, a key limitation of current approaches is that they tend to adopt a “snapshot” assessment of BP and lifestyle factors rather than capturing the dynamic trajectories of BP change and the temporal linkage with behavioural variables. Emerging evidence suggests that short-term changes in lifestyle behaviours and psychological stress may translate into measurable BP trends, which in turn may precede overt hypertension^11, 12^. For instance, higher perceived stress has been associated with increased BP variability and salt-sensitivity, potentially accelerating BP rise over time^13^. Similarly, repeated physical activity patterns and diet quality have demonstrated stronger associations with incident hypertension when studied longitudinally rather than cross-sectionally^14^.

From a predictive standpoint, there is growing emphasis on applying statistical and machine learning techniques to forecast BP trajectories using behavioural and clinical data. Such models enable early identification of individuals at risk of BP elevation, allowing for timely, personalized lifestyle interventions. By integrating dynamic measures of diet, physical activity, and psychological stress with repeated BP observations, these approaches can enhance precision in predicting short-term BP fluctuations and support proactive hypertension prevention strategies.

In this context, the present study examined the relationship between lifestyle and behavioural variables, including dietary patterns (sodium intake and diet quality), physical activity, and perceived stress, with short-term systolic blood pressure (SBP) trends among adults attending a preventive cardiology clinic. A regression-based predictive model was developed to estimate 12-week SBP and identify individuals at risk of BP elevation (≥5 mmHg). The study aimed to quantify the independent influence of modifiable behavioural predictors on SBP trajectory and to evaluate the predictive performance of a model integrating baseline and interim behavioural data.

## Materials and Methods

### Study design and setting

This was a prospective, longitudinal cohort study conducted over a period of three months in the outpatient cardiology and preventive cardiology departments of a tertiary-care teaching hospital. Participants attended three study visits at baseline, approximately six weeks, and twelve weeks. The design captured within-person changes in blood pressure while repeatedly measuring lifestyle and behavioral predictors.

### Ethical considerations

The Institutional Ethics Committee approved the study protocol before recruitment. Participant confidentiality was maintained by de-identifying all data. Participation was voluntary, and withdrawal from the study did not affect ongoing care.

### Sample size calculation

The sample size was estimated to evaluate longitudinal associations between behavioural predictors and short-term systolic blood pressure (SBP) trajectory in a repeated-measures framework. Assuming a small-to-moderate effect size (Cohen’s *f*^2^ = 0.10), two-sided α = 0.05, statistical power of 80%, and up to 10 candidate predictors, approximately 90 independent observations would be required in a conventional multivariable regression model.

However, because the present study involved repeated BP measurements within the same participants across three time points, observations were not fully independent due to within-subject correlation. Therefore, the repeated-measures structure was accounted for analytically using linear mixed-effects modelling rather than by inflating the nominal sample size using a conventional design effect formula.

Considering feasibility constraints and an anticipated attrition rate of approximately 20–25%, a total of 75 participants were enrolled. Of these, 67 participants completed follow-up and were included in the final analysis. Given the modest sample size relative to the number of predictors evaluated, the predictive modelling component should be interpreted as exploratory and internally validated, warranting future external validation in larger cohorts.

### Participants

Adults aged 18 to 70 years who were able to provide informed consent, owned a phone capable of receiving reminders, and were willing to complete lifestyle and stress questionnaires were included. Participants were required to have at least two prior clinic BP recordings in the preceding six months to establish baseline trends. Patients with secondary hypertension, pregnancy, unstable coronary or heart failure status in the preceding four weeks, end-stage renal disease on dialysis, or cognitive or psychiatric disorders that impaired the ability to complete questionnaires were excluded. Participants were recruited consecutively from the outpatient clinic. The study details were explained in the participant’s preferred language, and written informed consent was obtained prior to enrolment.

### Outcome measures

The primary outcome was the slope of systolic blood pressure (SBP) over time (mmHg per month), modelled from repeated measurements for each participant. Secondary outcomes included short-term changes in systolic and diastolic BP from baseline to twelve weeks and binary classification of BP trends as “rising” (≥5 mmHg increase) or “non-rising” (<5 mmHg increase).

### Blood pressure measurement protocol

BP was recorded using a validated automated oscillometric device (Omron HEM-907, Omron Healthcare Co., Kyoto, Japan). Measurements were taken in the morning after at least five minutes of rest, with participants seated, the arm supported at heart level, and having abstained from caffeine, exercise, or smoking for at least 30 min. Three consecutive readings were obtained at one-minute intervals, and the mean of the last two readings was recorded as the clinic BP. Additionally, participants measured home BP on three non-consecutive days (two readings in the morning and evening), and the averages were computed. Analyses were conducted using both clinic-only and combined clinic-plus-home BP data.

### Predictor variables

Dietary intake was assessed using a semi-quantitative Food Frequency Questionnaire (FFQ) adapted from previously evaluated Indian dietary assessment tools^15^. The sodium score was derived from the frequency of intake of high-salt foods, processed foods, salty snacks, pickles, and discretionary salt use. The score was intended as a pragmatic proxy measure of sodium-related dietary behaviour rather than a direct quantitative estimate of sodium intake. Because 24-hour urinary sodium excretion was not measured, some degree of exposure misclassification remains possible. DASH-like dietary adherence scores were also derived from FFQ responses to reflect overall dietary quality.

Physical activity was measured using the International Physical Activity Questionnaire–Short Form (IPAQ-SF), providing total metabolic equivalent (MET) minutes per week and sedentary time^16^. A subset of participants provided objective step-count data obtained through the Samsung Health mobile pedometer application (Samsung Electronics Co., Suwon, South Korea), which automatically records ambulatory movement using the device’s built-in accelerometer.

Participants were instructed to carry their smartphone throughout waking hours for at least seven consecutive days prior to each study visit. Step counts were retrieved from the app’s activity log and averaged across valid monitoring days (minimum of five days, including one weekend day)^17^. Perceived stress was evaluated using the Perceived Stress Scale (PSS-10), with scores categorized into low, moderate, or high stress levels^18, 19^. Clinical covariates included age, sex, body mass index (BMI), baseline BP, diabetes, dyslipidemia, smoking status, alcohol use, number of antihypertensive drug classes, statin use, and baseline eGFR.

### Data collection procedures

At the baseline visit, demographic details, medical history, height, weight, and clinic BP were recorded. Participants completed the FFQ, IPAQ-SF, and PSS-10 questionnaires and were instructed in home BP measurement and app-based step tracking. At six and twelve weeks, BP was remeasured, and questionnaires were re-administered. Medication changes were documented. All BP devices were calibrated monthly, and data entry accuracy was verified by random cross-checks. Ten percent of the data were double-entered to ensure reliability.

### Data management

Data were entered into electronic case report forms with built-in range and logic checks. Missing data were explored for patterns, and when missing at random, multiple imputation using chained equations (MICE) with 20 imputations was applied. Derived scores were passively imputed.

### Statistical analysis

Continuous variables were summarized as mean ± standard deviation (SD) or median (interquartile range [IQR]) depending on data distribution, while categorical variables were presented as counts and percentages. Baseline characteristics between participants with rising versus non-rising BP trajectories were compared using independent-samples t-tests, Mann–Whitney U tests, Chi-square tests, or Fisher’s exact tests as appropriate.

Longitudinal associations between lifestyle predictors and SBP trajectory were evaluated using linear mixed-effects models (LMMs) with participant-specific random intercepts to account for repeated observations over time. Fixed effects included time, sodium score, DASH adherence score, physical activity level, perceived stress score, age, BMI, and antihypertensive treatment status.

For predictive modelling, Elastic Net regression was employed to predict 12-week SBP using baseline and interim behavioural variables. Continuous predictors were standardized before model fitting. Model hyperparameters were optimized using repeated 10-fold cross-validation. Model performance was evaluated using cross-validated R^2^, root mean square error (RMSE), and mean absolute error (MAE). Because of the relatively small sample size, no independent hold-out dataset was created; therefore, predictive performance should be interpreted as internally validated.

To evaluate the incremental predictive contribution of behavioural variables beyond baseline clinical parameters, model performance was compared between:

(i) a baseline clinical model containing baseline SBP, age, sex, BMI, and antihypertensive use; and

(ii) an expanded model additionally incorporating sodium score, DASH adherence, physical activity, and perceived stress.

Multicollinearity among predictors was assessed using Pearson correlation coefficients and variance inflation factors (VIFs). Missing data were explored for patterns, and when data were considered missing at random, multiple imputation using chained equations (MICE) with 20 imputations was performed.

Sensitivity analyses included:

(i) restricting analyses to clinic BP measurements only,

(ii) excluding participants with medication changes during follow-up, and

(iii) stratification according to antihypertensive treatment status.

Subgroup analyses were conducted according to age group and baseline stress tertiles. All statistical analyses were performed using R software version 4.4.0 (R Foundation for Statistical Computing, Vienna, Austria). A two-sided *p*-value <0.05 was considered statistically significant.

**Table 1:** Baseline demographic, clinical, and behavioral characteristics (*n* = 67).

**Variable**	**Mean ± SD / n (%)**
**Age (years)**	44.6 ± 10.8
**Sex (Male)**	35 (52.2%)
**BMI (kg/m^2^)**	26.8 ± 3.7
**Smokers**	14 (20.9%)
**Diabetes mellitus**	21 (31.3%)
**Dyslipidemia**	28 (41.8%)
**Baseline SBP (mmHg)**	134.2 ± 12.6
**Baseline DBP (mmHg)**	83.5 ± 8.4
**Antihypertensive use (any)**	37 (55.2%)
**Mean sodium score (0–20)**	11.2 ± 3.1
**DASH diet adherence score (0–10)**	5.8 ± 2.1
**PSS-10 score (0–40)**	18.0 ± 5.6
**Physical activity (MET-min/week)**	2,380 (1,250–4,500)
**Sedentary time (min/day)**	370 ± 105
**Mean daily step count**	6,240 ± 1,980

## Results

### Participant characteristics

Table 1 shows the baseline characteristics of the 67 participants included in the final analysis. The mean age was 44.6 ± 10.8 years, and 52.2% were male. The average baseline SBP and DBP were 134.2 ± 12.6 mmHg and 83.5 ± 8.4 mmHg, respectively. Mean BMI was 26.8 ± 3.7 kg/m^2^, and 55.2% were on antihypertensive therapy. Median physical activity was 2,380 MET-min/week, while the mean sodium score and DASH adherence scores were 11.2 ± 3.1 and 5.8 ± 2.1, respectively, indicating moderate dietary quality and activity levels at study entry.

Table S1 shows the comparison of baseline demographic, clinical, and behavioural characteristics between participants who completed follow-up (*n* = 67) and those lost to follow-up (*n* = 8). Non-completers demonstrated slightly higher perceived stress scores, higher sodium scores, lower DASH adherence, and lower physical activity levels compared with completers, although these differences did not reach statistical significance (all *p* > 0.05). Baseline age, sex distribution, BMI, systolic and diastolic blood pressure, and antihypertensive treatment status were broadly comparable between groups, suggesting minimal systematic baseline differences between completers and non-completers. However, the observed behavioural trends among non-completers raise the possibility of mild retention bias, which should be considered when interpreting the study findings.

### Blood pressure trends over 12 weeks

**Figure 1.**
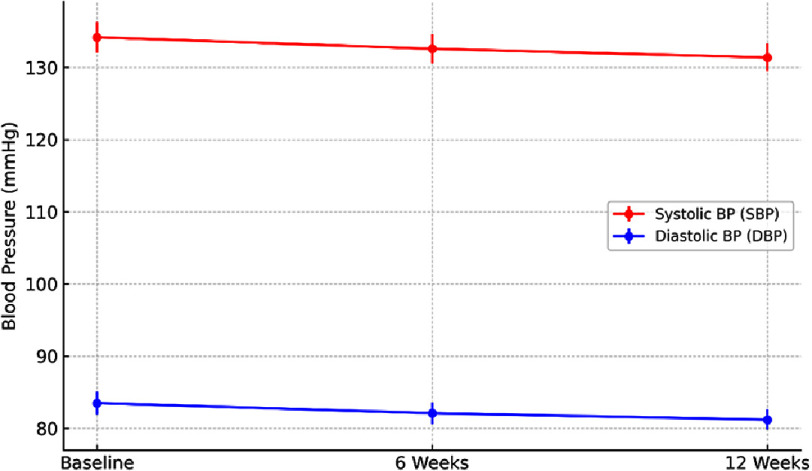
Mean systolic and diastolic blood pressure across follow-up visits.

Figure 1 shows the temporal trend of mean systolic and diastolic blood pressure (SBP and DBP) measured at baseline, 6 weeks, and 12 weeks. Both SBP and DBP exhibited a gradual downward trend across the 12-week follow-up period. At baseline, the mean SBP was 134.2 ± 12.6 mmHg, which changed to 132.6 ± 11.9 mmHg at 6 weeks and 131.4 ± 11.5 mmHg at 12 weeks. DBP decreased modestly from 83.5 ± 8.4 mmHg to 81.2 ± 7.6 mmHg at 12 weeks.

Overall, 41 participants (61.2%) exhibited a downward or stable BP trend (“non-rising group”), while 26 (38.8%) showed a rise in SBP ≥5 mmHg (“rising group”).

### Correlation analysis

Table 2 summarizes the bivariate correlations between lifestyle predictors and changes in SBP over 12 weeks. Higher sodium intake (r = 0.33, *p* = 0.007) and greater perceived stress (r = 0.36, *p* = 0.004) were positively correlated with SBP increase, while higher physical activity (r = −0.38, *p* = 0.002) and DASH diet adherence (r = −0.34, *p* = 0.005) were inversely correlated. Sedentary time showed a mild positive correlation (r = 0.28, *p* = 0.018).

**Table 2:** Pearson correlation between lifestyle factors and SBP change over 12 weeks.

**Predictor**	**r**	***p*** **-value**	**Direction**
Sodium score	+0.33	0.007	Positive
DASH adherence score	−0.34	0.005	Negative
PSS-10 stress score	+0.36	0.004	Positive
Physical activity (MET-min/week)	−0.38	0.002	Negative
Sedentary time	+0.28	0.018	Positive
Daily step count	−0.31	0.011	Negative

Table 3 presents the results of the mixed-effects regression model evaluating time-varying predictors of SBP trajectory. Over the 12-week period, SBP declined significantly (β = −1.15 mmHg/month, *p* = 0.001). Higher physical activity (β = −0.84, *p* = 0.003) and greater DASH diet adherence (β = −0.67, *p* = 0.006) were associated with reduced SBP, whereas higher sodium intake (β = +0.58, *p* = 0.012) and elevated stress (β = +0.71, *p* = 0.008) predicted rising SBP. The model explained 41% of the fixed-effect variance and 67% of total variance, indicating good fit.

**Table 3:** Linear mixed-effects model for predictors of systolic BP trend.

**Predictor**	**β (95% CI)**	**SE**	***p*** **-value**
Time (months)	−1.15 (–1.85 to –0.45)	0.34	0.001
Sodium score (per SD)	+0.58 (0.13–1.03)	0.22	0.012
DASH diet score (per SD)	−0.67 (–1.15 to –0.19)	0.24	0.006
Physical activity (per SD)	−0.84 (–1.40 to –0.28)	0.28	0.003
PSS-10 stress (per SD)	+0.71 (0.19–1.24)	0.26	0.008
BMI (kg/m^2^)	+0.21 (–0.05 to 0.46)	0.13	0.11
Antihypertensive use (yes)	−0.92 (–2.04 to 0.20)	0.56	0.10
Age (years)	+0.09 (0.01–0.17)	0.04	0.029

**Figure 2.**
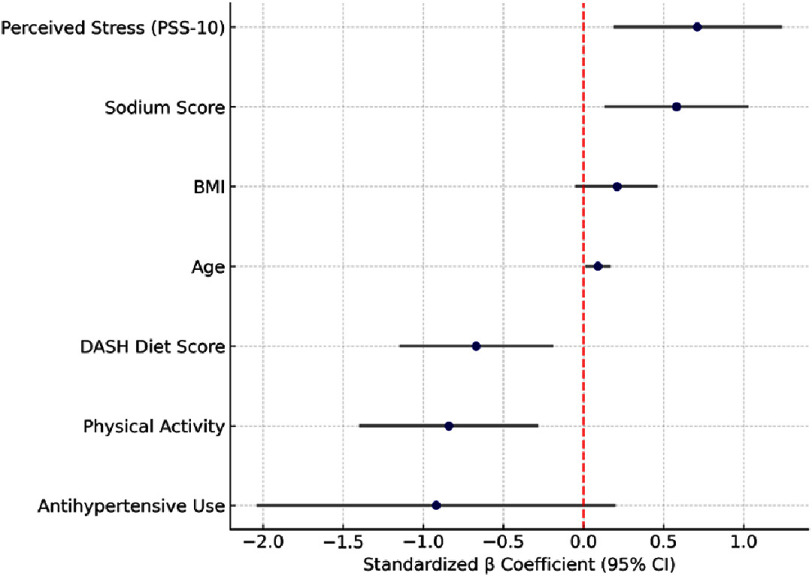
Forest plot of standardized coefficients for predictors of SBP trend.

Figure 2 presents a forest plot summarizing the standardized β-coefficients (95% CI) derived from the mixed-effects regression model for predictors of systolic BP trend. Among the lifestyle and behavioral variables, higher physical activity (β = −0.84, *p* = 0.003) and greater DASH diet adherence (β = −0.67, *p* = 0.006) were significantly associated with a reduction in SBP over time, indicating protective effects. Conversely, higher sodium intake (β = +0.58, *p* = 0.012) and higher perceived stress scores (β = +0.71, *p* = 0.008) predicted a rise in SBP. Age showed a minor positive association (β = +0.09, *p* = 0.029).

### Predictive model performance

Elastic Net regression was employed to predict 12-week systolic blood pressure (SBP) using baseline and 6-week variables. The optimized model retained six key predictors: baseline SBP, perceived stress score (PSS-10), sodium intake score, DASH diet adherence, physical activity level, and age. The model demonstrated excellent predictive accuracy, explaining 78% of the variance (*R*^2^ = 0.78), with a root mean square error (RMSE) of 5.9 mmHg and a mean absolute error (MAE) of 4.5 mmHg. Cross-validation confirmed stable performance with a mean cross-validated R^2^ of 0.74 ± 0.05, indicating strong generalizability and minimal overfitting.

**Figure 3.**
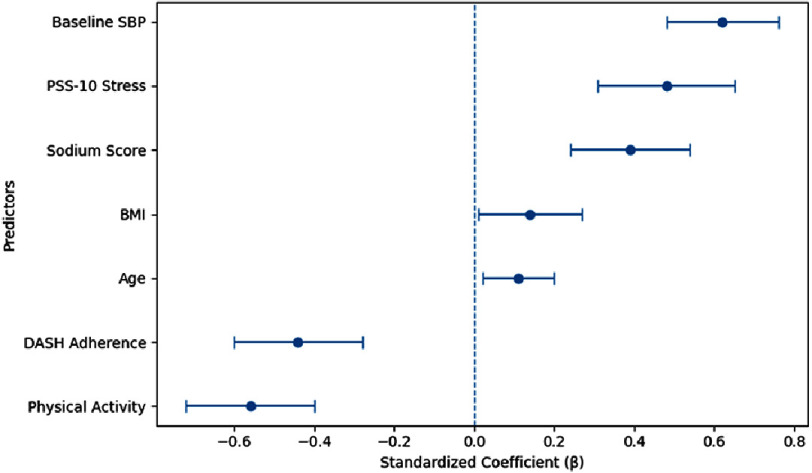
Standardized coefficient plot for Elastic Net regression model predicting 12-week systolic BP.

Figure 3 presents the standardized coefficient plot derived from the Elastic Net regression model used to predict 12-week systolic blood pressure (SBP). The plot summarizes the relative contribution and direction of association of the retained predictors within the model. Higher perceived stress (PSS-10) scores and higher sodium scores demonstrated positive associations with predicted SBP, indicating greater likelihood of BP elevation. In contrast, greater DASH diet adherence and higher physical activity levels showed negative associations, reflecting protective effects against SBP rise. Baseline SBP remained an important positive predictor, whereas age and BMI demonstrated comparatively smaller positive contributions to the model.

### Binary classification model

**Figure 4.**
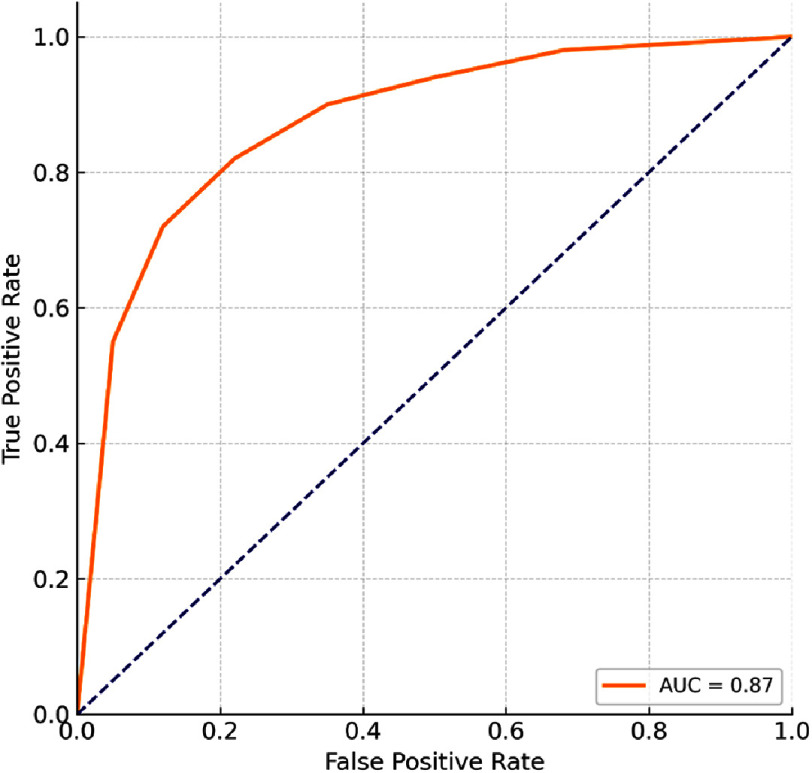
ROC curve for classification of rising vs. non-rising SBP. Plot showing AUC = 0.87, with diagonal reference line.

Figure 4 displays the Receiver Operating Characteristic (ROC) curve for the binary classification model distinguishing participants with rising (≥5 mmHg increase) versus non-rising SBP. The model demonstrated excellent discrimination with an AUC of 0.87 (95% CI [0.78–0.95]), sensitivity of 84%, and specificity of 79%, and Brier score 0.11 (good calibration). The curve lies well above the diagonal reference line, confirming the high accuracy and clinical utility of the model for early identification of individuals at risk of worsening blood pressure trends.

Table 4 shows the results of sensitivity and subgroup analyses confirming the robustness of the main model. When only clinic BP readings were analyzed, associations remained consistent though slightly attenuated (β =  − 0.73, *p* = 0.012). Excluding participants with medication changes (*n* = 5) did not affect outcomes (β =  − 0.82, *p* = 0.004), and substituting the sodium score with the DASH adherence score retained significance (β =  − 0.67, *p* = 0.006). In subgroup analyses, participants aged <45 years showed a stronger inverse relationship between physical activity and SBP (β =  − 1.02, *p* = 0.002) compared to those ≥45 years (β =  − 0.58, *p* = 0.045). Among highly stressed individuals (PSS-10 > 22), sodium intake amplified SBP rise (β =  + 0.90, *p* = 0.031), underscoring the modifying effects of age and stress on BP response to lifestyle factors.

**Table 4:** Sensitivity and subgroup analyses of predictors of systolic blood pressure change.

**Analysis type**	**Predictor/Condition**	**β (mmHg/ month)**	**95% CI**	***p*** **-value**
**Sensitivity 1**	Clinic BP readings only	−0.73	−1.29 to –0.17	0.012
**Sensitivity 2**	Excluding medication changes (*n* = 5)	−0.82	−1.38 to –0.26	0.004
**Sensitivity 3**	DASH diet score (replacing sodium)	−0.67	−1.15 to –0.19	0.006
**Subgroup 1**	Age < 45 years	−1.02	−1.65 to –0.39	0.002
	Age ≥ 45 years	−0.58	−1.13 to –0.03	0.045
**Subgroup 2**	High stress (PSS-10 > 22)	+0.90	+0.21 to +1.59	0.031
	Low–moderate stress (PSS-10 ≤ 22)	+0.43	+0.02 to +0.84	0.041

### Incremental predictive value of behavioural variables

To determine the additional predictive contribution of behavioural variables beyond baseline clinical factors, model performance was compared between nested Elastic Net models. A baseline clinical model including baseline SBP, age, sex, BMI, and antihypertensive treatment status achieved an R^2^ of 0.61 with an RMSE of 7.8 mmHg. Following the addition of behavioural variables including sodium score, DASH adherence score, physical activity, and perceived stress, predictive performance improved substantially to an R^2^ of 0.78 and RMSE of 5.9 mmHg. Thus, behavioural variables contributed an incremental R^2^ improvement of 0.17, supporting their independent predictive value in short-term BP trajectory estimation.

### Comparison of completers and non-completers

Baseline characteristics of participants who completed follow-up (*n* = 67) and those lost to follow-up (*n* = 8) were compared to evaluate potential retention bias. Non-completers demonstrated slightly higher perceived stress scores and lower physical activity levels than completers, although these differences did not reach statistical significance. Baseline SBP, age, sex distribution, and BMI were broadly comparable between groups. These findings suggest the possibility of mild behavioural retention bias, which should be considered when interpreting the results.

### Collinearity assessment

The FFQ-derived sodium score and DASH adherence score demonstrated a moderate inverse correlation (Pearson r = −0.46). Variance inflation factors remained low for both variables (sodium score VIF = 1.42; DASH adherence VIF = 1.39), indicating absence of substantial multicollinearity within the regression models.

## Discussion

In this prospective cohort study, we found that modifiable lifestyle and behavioural factors—specifically dietary sodium intake, diet quality (DASH adherence), physical activity, and perceived stress—were independently and robustly associated with short-term SBP trajectories over 12 weeks. The linear mixed-effects model demonstrated that higher physical activity (β = −0.84 mmHg/month), greater DASH diet adherence (β = −0.67 mmHg/month), higher sodium-intake scores (β = +0.58 mmHg/month), and higher stress (β = +0.71 mmHg/month) significantly influenced SBP slope. Our predictive Elastic Net model achieved *R*^2^ = 0.78 for 12-week SBP, and the classification model achieved AUC = 0.87 for identifying participants with SBP rise ≥5 mmHg. Sensitivity and subgroup analyses confirmed the stability of these associations across measurement modalities, medication changes, age strata, and stress levels. Overall, our findings reinforce and extend existing evidence linking behavioural determinants to BP trends and support the promise of relatively simple AI-augmented regression models for BP risk stratification.

The observed inverse association between physical activity and SBP slope aligns with a substantial body of literature demonstrating that greater physical activity levels reduce BP. A meta-analysis of 60 studies (n ≈ 11,000) reported a weighted mean difference in SBP of −7.70 mmHg (95% CI [−9.50 to −5.91 mmHg]) with physical activity interventions, compared with controls^20^. While our effect size is smaller (−0.84 mmHg/month translates to ∼−2.5 mmHg over 3 months), the short follow-up and inclusion of free-living behaviour rather than supervised exercise likely explain the difference. Importantly, the stronger effect in younger participants (<45 years: β = −1.02) suggests that earlier lifestyle modification may yield greater BP responsiveness, possibly due to less vascular stiffness or greater physiological reserve.

Diet quality and sodium intake also emerged as key determinants. Our finding of a positive association between sodium score and SBP slope is consistent with meta-analytic evidence indicating a linear dose–response relationship between dietary sodium and BP: one meta-regression found a reduction of −7.7 mmHg SBP per 100 mmol sodium reduction (≈2.3 g salt) in higher BP groups^21^. Another recent dose–response review confirmed that higher sodium intake is associated with increased risk of hypertension in prospective cohort data^22^. Moreover, a modest salt reduction (≥100 mmol/day) was associated with SBP reduction in long-term trials^23^. Our results extend this by showing that even short-term behavioural variation in sodium proxies corresponded to SBP trajectory, emphasising the dynamic nature of dietary influence on BP.

Diet quality (DASH adherence) had a protective effect, which aligns with the established benefits of the DASH dietary pattern. The DASH-Sodium trial found reductions of SBP/DBP when combining a DASH diet with low sodium in hypertensive participants^24^. Our β of −0.67 mmHg/month implies ∼−2.0 mmHg over three months from a 1-SD increase in DASH score, consistent with smaller magnitude due to shorter duration and “real-world” measurement. The substitution of DASH score for sodium in sensitivity analysis maintained statistical significance (β = −0.67, *p* = 0.006), reinforcing that diet quality beyond salt alone contributes to BP trend modulation.

The positive association between perceived stress (PSS-10) and SBP trajectory (β = +0.71 mmHg/month) emphasises the important role of psychological factors in BP dynamics. While evidence for stress and BP has been less consistent, observational studies in young adults have shown that higher perceived stress is associated with poorer BP control^25^. Mechanistically, stress may promote sympathetic activation, endothelial dysfunction, and salt sensitivity, combining with dietary/behavioural factors to accelerate BP rise.

Our predictive modelling results are encouraging: R^2^ of 0.78 and MAE 4.5 mmHg over 12 weeks indicate that a relatively modest set of behavioural and clinical variables can predict short-term SBP with good accuracy. The classification model’s AUC = 0.87 suggests strong ability to identify individuals likely to experience an adverse BP trend. These findings support the potential translation of regression/AI-augmented tools into clinical or community settings for early identification of individuals at risk of BP worsening, facilitating targeted lifestyle interventions^26, 27^.

Historically, long-term hypertension risk has been linked to lifestyle factors such as diet, physical activity, BMI and alcohol^28, 29^. However, fewer studies have focused on short-term BP trajectories in relation to behavioural variables, especially in cohort data. Our study fills this gap by demonstrating that behavioural fluctuations within a 12-week window are meaningfully associated with BP slopes. While large-scale cohort studies such as the INTERSALT have linked 24-h urinary sodium and BP cross-sectionally^29^, ours is among the first to integrate stress, diet, activity and short-term BP change, and then apply predictive modelling.

The stronger physical activity effect observed among younger participants aligns with existing evidence suggesting that younger vascular systems exhibit greater responsiveness to lifestyle modification. Longitudinal data indicate that higher cardiorespiratory fitness maintained over time is associated with a substantially lower risk of developing hypertension in later adulthood^30, 31^. The interaction between stress and sodium intake (synergistic effect in high-stress group) is a novel finding: stress may amplify salt sensitivity and BP variability. This bears similarity to work on salt sensitivity being higher in those with sympathetic overactivity or chronic stress, though direct prior data are limited^32, 33^.

Our results have several important implications. They indicate that even short-term monitoring of behavioural factors can meaningfully contribute to blood pressure (BP) risk stratification. Clinicians and public health programmes could incorporate simple validated questionnaires such as the PSS-10, dietary FFQ, and IPAQ-SF alongside routine BP measurement to identify individuals at risk of rising BP. The modifiable nature of these predictors including diet, physical activity, and stress provides clear and actionable targets for intervention. Behavioural salt-reduction programmes and physical activity promotion have been consistently shown to lower systolic BP by several millimetres of mercury, underscoring the value of addressing these factors for effective population-level hypertension prevention.

Moreover, the predictive model may allow pre-emptive lifestyle “nudges”: patients flagged at higher risk of BP rise can receive intensified counselling or follow-up. In resource-limited settings, such as rural India, this approach may help triage high-risk individuals for community health workers or NHM (National Health Mission) screening. Early intervention may prevent progression from high-normal BP to overt hypertension.

### Strengths and limitations of the study

Strengths of this study include the prospective design with repeated BP measurements (clinic + home), use of validated lifestyle and stress instruments (IPAQ-SF, PSS-10, FFQ), application of both traditional and machine-learning-type predictive modelling, and sensitivity/subgroup analyses to assess robustness. The high retention (89.3%) and integration of home BP readings strengthen ecological validity.

However, the present study should be interpreted in light of several limitations. First, the final analytical sample of 67 participants was relatively modest for multivariable predictive modelling; therefore, the model should be considered exploratory and internally validated. Second, antihypertensive treatment was categorized mainly as treated versus untreated without detailed assessment of drug class, dosage, or adherence, and residual medication-related confounding may persist. Third, dietary sodium intake was estimated using an FFQ-derived proxy score rather than 24-hour urinary sodium excretion, which may have introduced exposure misclassification. Fourth, non-completers demonstrated slightly higher stress and lower physical activity levels, suggesting possible mild retention bias. Finally, the single-centre design and short follow-up duration may limit generalisability, and external validation in larger cohorts is required.

Further research should extend follow-up to 12 months or more to examine whether the short-term trends we observed translate into sustained BP changes, incident hypertension, or cardiovascular outcomes. External validation of the predictive model in diverse settings is essential. Incorporating additional variables such as sleep duration/quality, salt-sensitivity genotype, wearable-device captured physical activity/HRV, and ecological momentary stress assessment may enhance predictive power. From a public health perspective, implementation studies are needed to test whether lifestyle-based prediction and early intervention (via mobile health or community health workers) reduce incident hypertension and cardiovascular events.

## Conclusion

In conclusion, this prospective cohort study demonstrated that lifestyle and behavioural metrics—sodium intake, diet quality, physical activity and perceived stress—independently predicted short-term systolic BP trajectory and could be integrated into a high-performing predictive model. These findings underscore the dynamic nature of BP regulation, highlight the potential for early lifestyle-based risk stratification, and support the incorporation of simple behavioural assessments into BP management and prevention programmes. The results offer actionable targets for clinicians and public-health practitioners and set the stage for scalable AI-augmented models to personalise hypertension prevention.
